# Synchrony in Networks of Type 2 Interneurons Is More Robust to Noise with Hyperpolarizing Inhibition Compared to Shunting Inhibition in Both the Stochastic Population Oscillator and the Coupled Oscillator Regimes

**DOI:** 10.1523/ENEURO.0399-23.2024

**Published:** 2024-03-25

**Authors:** Roman Baravalle, Carmen C. Canavier

**Affiliations:** Department of Cell Biology and Anatomy, Louisiana State University Health Sciences Center-New Orleans, New Orleans, Louisiana 70112

**Keywords:** chloride ions, fast spiking interneurons, GABA receptors

## Abstract

Synchronization in the gamma band (25–150 Hz) is mediated by PV+ inhibitory interneurons, and evidence is accumulating for the essential role of gamma oscillations in cognition. Oscillations can arise in inhibitory networks via synaptic interactions between individual oscillatory neurons (mean-driven) or via strong recurrent inhibition that destabilizes the stationary background firing rate in the fluctuation-driven balanced state, causing an oscillation in the population firing rate. Previous theoretical work focused on model neurons with Hodgkin's Type 1 excitability (integrators) connected by current-based synapses. Here we show that networks comprised of simple Type 2 oscillators (resonators) exhibit a supercritical Hopf bifurcation between synchrony and asynchrony and a gradual transition via cycle skipping from coupled oscillators to stochastic population oscillator (SPO), as previously shown for Type 1. We extended our analysis to homogeneous networks with conductance rather than current based synapses and found that networks with hyperpolarizing inhibitory synapses were more robust to noise than those with shunting synapses, both in the coupled oscillator and SPO regime. Assuming that reversal potentials are uniformly distributed between shunting and hyperpolarized values, as observed in one experimental study, converting synapses to purely hyperpolarizing favored synchrony in all cases, whereas conversion to purely shunting synapses made synchrony less robust except at very high conductance strengths. In mature neurons the synaptic reversal potential is controlled by chloride cotransporters that control the intracellular concentrations of chloride and bicarbonate ions, suggesting these transporters as a potential therapeutic target to enhance gamma synchrony and cognition.

## Significance Statement

Brain rhythms in the gamma frequency band (25–150 Hz) depend on the activity of inhibitory interneurons and evidence for a causal role for gamma oscillations in cognitive functions is accumulating. Here, we extend previous studies on synchronization mechanisms to interneurons that have an abrupt threshold frequency below which they cannot sustain firing. In addition to current-based synapses, we examined inhibitory networks with conductance-based synapses. We found that if the reversal potential for inhibition was below the average membrane potential (hyperpolarizing), synchrony was more robust to noise than if the reversal potential was very close to the average potential (shunting). These results have implications for therapies to ameliorate cognitive deficits.

## Introduction

Synchrony in the gamma frequency band [25–150 Hz (Colgin et al., 2009)] is critical to cognition and disrupted in Alzheimer's disease (Sahu and Tseng, 2023) and schizophrenia (Sohal, 2022), giving rise to cognitive deficits. Rhythmic stimulation at gamma frequency (Lakatos et al., 2019) is being utilized as a putative therapeutic intervention for cognitive impairment. In order to develop effective and precisely targeted therapeutics, it is imperative to determine the mechanisms underlying gamma synchrony.

Excitable neurons in general can fire action potentials in two modes. One is a pacemaker-like mode in which the input to the neuron is generally above threshold and the firing rate is determined by the mean current input, often called the mean-driven regime in contrast to a fluctuation-driven regime (Schreiber et al., 2009; Petersen and Berg, 2016). Networks of coupled oscillatory neurons in the mean-driven regime were discovered to be capable of synchrony mediated by inhibitory synapses (Van Vreeswijk et al., 1994), without the need for excitatory synapses. An influential study showed (Wang and Buzsáki, 1996) that inhibitory synchrony was not robust to the levels of heterogeneity thought to characterize physiological networks. In a seminal series of papers (Brunel and Hakim, 1999; Brunel and Hansel, 2006), Brunel and colleagues analyzed networks of inhibitory neurons and discovered a type of synchrony mediated by inhibition that did not depend on coupling between oscillatory neurons, but instead arose from the interactions between population rate and the synaptic inhibition recruited by that rate. In the mean-driven regime, one would expect the distribution of interspike intervals to be Gaussian. In the fluctuation-driven regime, the neuron is presumed to be in a state with balanced excitation and inhibition (Shadlen and Newsome, 1998) biased slightly below the threshold for action potential generation. The balanced excitation and inhibition produce fluctuations that provide a diffusive drive in the presence of a drift back toward the resting membrane potential. This results in a mean-reverting random walk (Uhlenbeck and Ornstein, 1930) in the membrane potential (Brunel and Hakim, 1999). The firing of individual neurons appears random and the distribution of interspike intervals (ISI) is exponential except for a refractory period. Networks in the fluctuation driven regime produce stochastic population oscillations for sufficiently large noise and sufficiently strong inhibition.

The original theoretical work described above used only integrators (Izhikevich, 2000) with Hodgkin's Type 1 excitability (Hodgkin, 1948), meaning that they can fire at arbitrarily low rates, and studied only current based synapses. The bifurcation structure defines the difference between Type 1 and Type 2 excitability (Ermentrout, 1996; Rinzel and Ermentrout, 1998). We have previously shown that PV+ interneurons in layer 2/3 of medial entorhinal cortex exhibit Type 2 excitability (Tikidji-Hamburyan et al., 2015), meaning that there is a cutoff frequency below which repetitive firing cannot be sustained. We systematically explored the responses of resonator neurons (Izhikevich, 2001) with Hodgkin's Type 2 excitability. We identify two routes to the stochastic population oscillator (SPO), similar to the previously observed routes in Type 1 neurons (Brunel and Hansel, 2006), except that that Type 2 excitability endows neurons with postinhibitory rebound (Perkel and Mulloney, 1974; Wang and Rinzel, 1993; Rinzel et al., 1998; Tikidji-Hamburyan et al., 2015). The first route occurs via supercritical Hopf bifurcation from the stationary asynchronous mode, in which the firing rate is approximately constant, into an SPO. The other route is a transition from the coupled oscillator mode to the SPO (Tikidji-Hamburyan et al., 2015). We also studied a biophysically calibrated (Fernandez et al., 2022; Via et al., 2022) model of a network of PV+ interneurons in layer 2/3 of medial entorhinal cortex with conductance rather than current based synapses with either shunting or hyperpolarizing synapses (Vida et al., 2006), or a uniform distribution of reversal potential between the two extremes. Whereas synchrony was more robust in networks with hyperpolarizing synapses compared to shunting or a uniform distribution, the uniform distribution only outperformed shunting below a threshold for synaptic connection strength.

## Materials and Methods

### Izhikevich Type 2 model

The equations describing the dynamics of an Izhikevich resonator neuron model are as follows:

(1) $$C_{m}\frac{dV(t)}{dt}=0.04\;V^{2}(t)+5\;V(t)+140-u(t)+I(t)=f_{\mathrm{Izhi}}(V,u)+I(t),$$

(2) $$\frac{du(t)}{dt}=a\;(b\;V(t)-u(t)).$$

If $V>v_{\mathrm{peak}}$, then V←c,u←u+d. We use the same values as in our previous work (Tikidji-Hamburyan et al., 2015). The parameters values are *a* = 0.1 ms^−1^, *b* = 0.26 nS, *c* = −65 mV, *d* = 0 nA. *C_m_* is 1 µF/cm^2^. Currents are in nA/cm^2^.

Postspike adaptation was neglected with parameter d set to zero. As the parameter b is positive, this model exhibits Type 2 excitability, with a discontinuous *f*-*I* curve.

### Via PV+ fast spiking interneuron model

We also use a calibrated Hodgkin–Huxley type conductance-based model with Type 2 excitability. The model is described in detail in (Via et al., 2022). This single compartment model neurons has five state variables: the membrane potential (*V*) and four gating variables (*m*, *h*, *n*, and *a*) that use the same kinetic equations as the original Hodgkin–Huxley model (Hodgkin and Huxley, 1952; Baxter et al., 2004), but with different parameters tuned to replicate the dynamics of fast spiking neurons in the medial entorhinal cortex. We included two delayed rectifier *K*^+^ currents (*I*_Kv7_ and *I*_Kv3_). *K*_V_7 was mislabeled as *K*_V_1 in (Via et al., 2022). The differential equation for the membrane potential (*V*) of each neuron with this correction is now as follows:

(3) $$C_{m}\frac{dV}{dt}=I_{\mathrm{Na}}+I_{Kv7}+I_{Kv3}+I_{L}+I(t)=f_{\mathrm{Via}}(V,m,h,n,a)+I(t),$$

where *C_m_*_ _= 81.4 pF is the membrane capacitance, *I(t)* is the external current, *I*_Na_ is the fast sodium current, and *I_L_* is the passive leak current. The equations for the intrinsic ionic currents are as follows: $I_{\mathrm{Na}}=g_{\mathrm{Na}}m^{3}h(E_{\mathrm{Na}}-V)$, $I_{Kv7}=g_{Kv7}a^{4}(E_{K}-V),\;I_{Kv3}=g_{Kv3}n^{4}(E_{K}-V)$, and $I_{L}=g_{L}(E_{L}-V)$, with *E*_Na _= 50 mV, *E_K_*_ _= −90 mV, *E_L_*_ _= −77.8 mV, *g_L_*_ _= 13.8 nS, *g*_Na _= 18,929 nS, *g*_KV7 _= 58.5 nS and *g*_kV3 _= 784.5 nS. The dynamics of the gating variables are given by $dx/dt=\alpha_{x}(1-x)-\beta_{x}x$ for the activation variables (*m*, *n*, *a*) and by $dx/dt=\beta_{x}(1-x)-\alpha_{x}x$ for the inactivation variable h, where $\alpha_{x}=k_{1x}(\theta_{x}-V)/(\exp\;((\theta_{x}-V)/\sigma_{1x})-1)$ and $\beta_{x}=k_{2x}\exp\;(V/\sigma_{2x})$ using parameters in Table 1.

**Table 1.** Parameters for gating variables

	*m*	*h*	*n*	*a*
Θ (mV)	−47.95	−49.72	11.32	42.85
σ_1_ (mV)	4	−20	12	12
σ_2_ (mV)	−13	3.5	−8.5	−80
*k*_1_ (ms^−1^)	0.25	0.012	1	1
*k*_2_ (ms^−1^)	0.1	0.2	0.001	0.02

### Homogeneous networks

We consider a fully connected network of *N* inhibitory neurons as *N* was varied from 800 to 3,000. In the subthreshold range $\left(V\leq V_{t}\right)$, where *V_t_* is the firing threshold, the membrane potential has the following dynamics:

(4) $$C_{m}\frac{dV_{i}}{dt}=f(V_{i},\{x\})+I(t)=f(V_{i},\{x\})+I_{\mathrm{ext}}(t)+I_{i,\mathrm{rec}}(t)+I_{i,\mathrm{noise}}(t),$$

where *f*(*V_i_*,{*x*}) is the function describing the single neuron membrane dynamics, where {*x*} stands for the gating variables (see Eq. 1 for Izhikevich model and Eq. 3 for Via model), and *C_m_* is the membrane capacitance of the model. Here, *I*_ext_ is a constant external input, *I_i_*_,noise_(*t*) is a noisy external input and *I_i_*_,rec_(*t*) is the recurrent input due to the interactions between the neurons.

The noisy current is modeled as $I_{i,\mathrm{noise}}(t)=\sigma \;\eta_{i}(t)$. Here, $\eta_{i}(t)$ is a white noise uncorrelated from neuron to neuron and from time to time. We define the noise intensity as σ (in nA/cm^2^ for Izhikevich model and nA for the PV+ FS model).

Since the network is fully connected, the recurrent synaptic input is the same for all neurons:

(5) $$I_{i,\mathrm{rec}}(t)=\frac{J}{N}\sum_{\;j=1}^{N}\sum_{k}s(t-t_{j}^{k}),$$

where *J* is the coupling strength which scales the postsynaptic current for a single synapse (current is given in nA/cm^2^ for Izhikevich model and in nA for Via model), *N* is the network size and $s(t-t_{j}^{k})$ denotes the postsynaptic current (PSC) elicited by a presynaptic spike in neuron j occurring at time $t_{j}^{k}$.

For the conductance-based model, the righthand side of Equation 5 is multiplied by the driving force, current is in nA and *J* is given in nS:

(6) $$I_{i,\mathrm{rec}}(t)=\frac{J}{N}\sum_{\;j=1}^{N}\sum_{k}s(t-t_{j}^{k})(V_{i}-E_{\mathrm{syn}}).$$

*E*_syn_ is the synaptic reversal potential. We ran simulations with purely hyperpolarizing (*E*_syn _= −75 mV) or shunting (*E*_syn _= −55 mV) synapses, and for the case in which *E*_syn_ was uniformly distributed between these two values. The first sum is over synapses, whereas the second sum is over spikes. We modeled the PSC function as a biexponential with latency $\tau_{L}$ = 1 ms, rise time constant $\tau_{R}$ = 1 ms, and decay time constant $\tau_{D}$ = 6 ms.

(7) $$s(t)=\{\begin{array}{ccc} 0 & \mathrm{if} & t<\tau_{L}\\ \frac{1}{\tau_{D}-\tau_{R}}\left(e^{\frac{t-\tau_{L}}{\tau_{D}}}-e^{\frac{t-\tau_{L}}{\tau_{R}}}\right) & \mathrm{if} & t\geq \tau_{L} \end{array}.$$

The factor $\left(1/(\tau_{D}-\tau_{R})\right)$ ensures that the integral of the PSC (i.e., the total charge received in the postsynaptic neuron due to a presynaptic spike) is one, i.e., ∫s(t)dt=1. For conductance-based synapses the maximum of *s*(*t*) is normalized to 1 (Via et al., 2022)

#### Heterogeneous network

A biophysically realistic heterogeneous network was constructed only for the conductance-based Via model with a 3D spatial structure corresponding to a mouse brain slice of layer 2/3 medial entorhinal cortex (mEC) measuring 800 µm × 300 µm × 800 µm. Based on the measured PV+ cell density in layers 2/3 of mEC (Bjerke et al., 2021), we assigned 321 neurons to the slice and positioned them randomly. The full extent of the experimentally measured variability across the PV+ population in terms of their passive properties and their frequency current relations was captured in our previously published 100 neuron model (Via et al., 2022). We used 50 unique neurons and cloned them as necessary to construct the 321 neuron heterogeneous network. Gabaergic synaptic connectivity was calibrated according to (Fernandez et al., 2022) in which the connection probability was found to be 36% in each direction inside a radius of 150 µm with a lognormal distribution of the synaptic conductances with a mean of *J* × 1.65 nS (Via et al., 2022), where 1.65 nS was the measured average synaptic conductance. The standard deviation 1.56 nS of the synaptic conductances used here was less than that used in (Via et al., 2022). However, that study assumed strong electrical synapses in order to obtain synchronous activity, and we neglect electrical synapses here. Synaptic delays were assigned with the observed range (Fernandez et al., 2022) and depend linearly on the distance inside a 150 µm radius, from 0.4 to 1.2 ms.

#### Prediction of Hopf bifurcation surface using mean field theory

Brunel and colleagues developed a clever method to find the parameter sets at which an oscillation becomes possible. They used a mean field approximation to the stationary state in which each neuron receives the same oscillation in synaptic current, and each synapse receives action potential inputs at the same frequency as the oscillation in current. In order for an oscillation at a given angular frequency ω to exist, the oscillation in the firing rate must have the same phase and amplitude after transformation to an oscillation in current by the biexponential synapse and subsequently back into an oscillation in rate by the individual neurons receiving the oscillatory synaptic input. A linear time invariant system is a cascade of linear operators that scale the amplitude of the input signal via multiplication by |H(ω)| and shift the phase by ∢H(ω). These quantities can be calculated for a linear system, but the transfer function H(ω) must be measured at each frequency for a nonlinear system. At a given level of external bias current and noise, the frequency at which the Hopf bifurcation occurs was determined by the value at which all the phase lags around the circuit summed to −360°. The phase lag for the synaptic latency is $∢H_{L}(\omega )=\omega \tau_{L}$, where $\tau_{L}$ is the latency. The phase lags for the rising and decaying exponentials are $∢H_{R}(\omega )=\arctan\;(-\omega \tau_{R})$ and $∢H_{D}(\omega )=\arctan\;(-\omega \tau_{D})$, where $\tau_{R}$ and $\tau_{D}$ are the rising and decaying time constants, respectively. Since the firing rate is positive but the synaptic current causes a negative change in the rate, an additional −180° phase lag ensues. The phase lag for the neural model $∢H_{N}(\omega )$ must be calculated numerically using simulations of a single neuron for each pair of σ and *I*_ext_ values by applying a sinusoidal input with *J* = 1 then measuring the phase lag of the noisy sinusoidal output. We made predictions at constant firing rate, consistent with previous work (Brunel and Hansel, 2006). The constant current *I*_ext_ was adjusted using a bisection search method, to obtain a constant average firing rate (with an error of 0.5 Hz) for every value of noise intensity σ, coupling strength *J* and network size *N*. Since the input is noisy, we found *I*_ext_ by averaging over 3 s of simulation, after discarding the initial 1 s transient.

Next we found the response of the neuron to a sinusoidal input with amplitude 10% of the external bias current to mimic a small sinusoidal perturbation to a steady asynchronous state, with the average frequency fixed at 17 or 30 Hz. Frequency of the input varied between 1 and 250 Hz, with a 2 Hz step. The simulations were run for 50 s and averaged over 20,000 realizations. To find the linear gain and the linear phase shift introduced by the neuron between the oscillatory input current to the oscillation in rate output, we used the phase and amplitude of the discrete Fourier transform for the peak output frequency.

Finally, the synaptic strength *J* was adjusted to ensure that the amplitudes of the rate and current waveforms were unchanged from cycle to cycle. The amplitude scale factor for a biexponential synapse with unitary conductance strength is as follows:

$$|H_{s}(\omega )|=\sqrt{\frac{1}{\left(1+\omega^{2}\tau_{R}^{2})(1+\omega^{2}\tau_{D}^{2}\right)}}.$$

The scaling factor$\left|H_{N}(\omega )\right|$ for the neural models was calculated using the same method to get the phase lag. Since the connection strength scales the output of the synaptic current, *J* was set according to the following:

$$|H_{N}(\omega )|=\frac{1}{J|H_{S}(\omega )|}.$$

### Network simulations

Parameter sweeps were performed on the high-performance computing cluster Tigerfish using BRIAN software (Stimberg et al., 2019). We varied the three parameters *J*, σ, and *I*_ext_ independently for each of four network sizes (*N* = 800, 1,400, 2,200, 3,000).

### Participation measure

In order to differentiate between SPOs and coupled oscillator regimes, we used a method developed by our group based on the cycle by cycle population period, as defined by the peaks of the population histogram (Tikidji-Hamburyan et al., 2015). The spikes were binned in 1 ms windows and a pulse with the height determined by the number of spikes in the bin was placed at the center of the bin. The resultant pulse train was low pass filtered by convolution with a Gaussian kernel with standard deviation of 10 ms and a length of 100 ms, which produced a time series with clear peaks in the network activity to use as a clock to compute the average level of participation in the oscillation, which we track as the average number of spikes per cycle (SPC) normalized by the population size. Note that random peaks of spike rate can be detected in finite networks even in the absence of network oscillations, for example, in random firing or in phase dispersion. In this case, the SPC still reflects the mean firing rate of the population. Values of SPC < 0.6 for a SPO and SPC > 0.9 for coupled oscillator (CO) and postinhibitory rebound (PIR) regions matched the regions in which an exponential versus a Gaussian distribution of the ISI was observed. Intermediate values corresponded to a transitional oscillatory region with subharmonic peaks in the distribution (for 10 ms bin width). For SPC > 0.9, we distinguished PIR from CO for regions in which the average instantaneous current (external bias plus recurrent input) remained below the Hopf bifurcation for the individual neuron at all times.

### Synchrony measures

We used a synchrony measure to quantify the boundary between synchrony in our simulations in order to test our mean field predictions. Since synchrony is related to the fluctuations of global variables, it can be defined by averaging these fluctuations over a long time (Hansel and Sompolinsky, 1992; Golomb and Rinzel, 1993, 1994; Ginzburg and Sompolinsky, 1994). The average membrane potential across a population of neurons at time *t* is as follows:

$$\bar{V}(t)=\frac{1}{N}\sum_{i=1}^{N}V_{i}(t).$$

The average over time, or expectation value, of the population-averaged membrane potential is $\left\langle \bar{V}(t)\right\rangle$. In a completely asynchronous system, any allowable value of V(t) has the same probability for an individual neuron across time as for single neurons across the population. Since these probability distributions are equal, the expectation value of their difference is zero. This makes the expectation value of the population variance $\sigma_{V}^{2}={\left(\bar{V}(t)-\left\langle \bar{V}(t)\right\rangle \right)}^{2}$ also zero. As a measure of synchrony (Brunel and Hansel, 2006), we normalized the population variance to the mean value of the variance $\left(\sigma_{V_{i}}^{2}={\left(\bar{V_{i}}(t)-\left\langle \bar{V_{i}}(t)\right\rangle \right)}^{2}\right)$ of single-cell membrane potentials *V_i_*(*t*):

(8) $$\chi (N)=\sqrt{\frac{\sigma_{V}^{2}}{(1/N)\sum_{i=1}^{N}\sigma_{V_{i}}^{2}}}.$$

Thus, for a fully asynchronous system χ(N)=0. In a completely synchronized system, the expectation value of the population variance is nonzero. Since all neurons have identical activity, the population variance (top term in Eq. 8) is equal to the average expectation value of the individual variance of each neuron (bottom term in Eq. 8), thus χ(N)=1. For less than perfect synchrony, the degree of synchrony can be quantified by the measure above. The central limit theorem implies that for an infinite network, N→∞, the synchrony measure behaves as follows:

(9) $$\chi (N)=\chi_{\infty }+\frac{\delta_{\chi }}{\sqrt{N}}+O\left(\frac{1}{N}\right),$$

where $\chi_{\infty }$ is the large *N* limit of χ(N) and $\delta_{\chi }$ measures the finite size correction to χ at the leading order.

For each network size *N*, we can simulate a network for a given noise intensity, coupling strength and external bias current and calculate the synchrony measure χ(N). After simulating several network sizes, we can fit the parameter $\chi_{\infty }$ using Equation 9. The set of parameters *J*, σ and *I*_ext_ that makes $\chi_{\infty }=0$ define the Hopf bifurcation manifold, which is the border between synchrony and asynchrony. At each value of *J* and *I*_ext_, we find the critical value of σ*_C_* corresponding to the Hopf bifurcation by plotting the values $\chi_{\infty }$as a function of σ and noting the point at which $\chi_{\infty }$ deviates from zero. At constant *J* and *I*_ext_, the following mathematical function describes the dependence of $\chi_{\infty }$ on σ:

(10) $$\chi_{\infty }=\{\begin{array}{ccc} A\;{\left(\sigma_{C}-\sigma \right)}^{1/2} & \mathrm{if} & \sigma \leq \sigma_{C}\\ 0 & \mathrm{if} & \sigma \geq \sigma_{C} \end{array},$$

where $\sigma_{C}$ is the critical noise at the Hopf bifurcations, for a given *J* and *I*_ext_. This method allowed us to find the surface corresponding to the Hopf bifurcation in the 3D space.

We contrasted this method of quantifying synchrony with one developed by our group based on the cycle by cycle population period, as defined by the peaks of the population histogram (Tikidji-Hamburyan et al., 2015) and described above in the *Participation Measure* section. Peaks were detected using downward zero crossings of the first derivative of the smoothed signal. The peak at the beginning of each network cycle was assigned a phase value of 0 and, at the end, a phase value of 2π. Each spike was assigned a phase depending upon where it fell within the cycle and a vector length of 1 starting from the origin. Then the vector sum of all the spike vectors constructed in this fashion was taken and normalized by the number of vectors, resulting in a vector with an average phase and a length between 0 and 1. This measure quantifies the level of synchrony of the individual neurons with the population rhythm but gives no information regarding the average level of participation in the oscillation, which we track separately as the average number of spikes per cycle (SPC) normalized by the population size. Note that random peaks of spike rate can be detected in finite networks even in the absence of network oscillations, for example, in random firing or in phase dispersion. In this case, the vector length is close to 0, but the SPC still reflects the mean firing rate of the population. The advantage of the vector length measure is that only the population raster plot is required to calculate synchrony and participation therefore we used this measure for the heterogeneous, finite size network.

### Software availability

https://github.com/RomanB22/NoisyInhibitoryNetwork.git

## Results

### Transitions to SPO in Type 2 model with current based synapses

**Figure 1.**
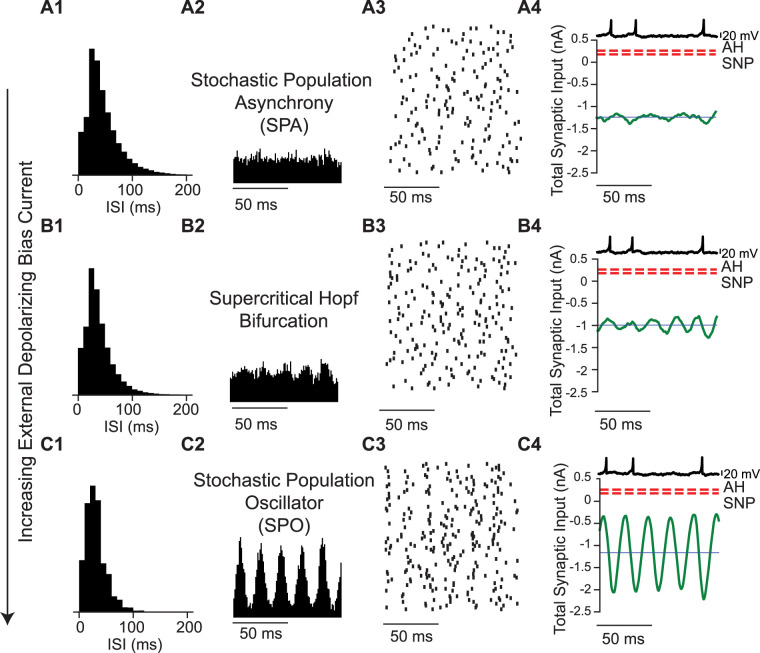
Transition from stochastic population asynchrony to SPO in a Type 2 neuron with increasing bias current. ***A***, Stochastic population asynchrony (*I*_ext _= 0.4 nA). ***A*1**, ISI histogram. ***A*2**, Spike time histogram. ***A*3**, Raster plot (down sampled 71 neurons out of 3,000). ***A*4**. Current relative to bifurcations [red dashed lines, AH Andropov–Hopf, SNP saddle node of periodics, total synaptic input current averaged over the network (green), and mean current for single neurons (dark blue)]. Single neuron voltage trace (light blue). ***B***, Emergence of SPO at the Hopf bifurcation (*I*_ext _= 1 nA). ***B*1–4** as in ***A*1–4**. Spikes per cycle (SPC) is 0.49. ***C***, Beyond the Hopf, the oscillation amplitude increases (*I*_ext _= 1.6 nA). ***C*1–4** as in ***A*1–4**. In all the three cases the synaptic strength is 7.94 nA and the noise intensity is 3.16 nA. SPC is 0.59. All units are given per cm^2^.

In order to determine whether our model neurons are in the mean driven or fluctuation driven regime, we first need to understand the bifurcation structure of Type 2 neurons that produce hysteresis in the frequency/current relationship. There is a bistable range of injected currents for which either repetitive firing or quiescence can be observed, depending upon whether the external bias current is stepped up such that the current steps become more depolarizing or stepped down. In the Izhikevich Type 2 model, there is a subcritical Andronov–Hopf (AH) bifurcation at *I* = 0.2625 nA/cm^2^, and there is a saddle node of periodics (SNP) bifurcation at *I* = 0.1795 nA/cm^2^. The AH is simply the threshold at which repetitive firing begins as the injected current becomes more depolarizing, and the SNP is simply the threshold below which repetitive firing (minimum value 27 Hz) cannot be sustained as the injected current becomes less depolarizing. The AH and SNP are marked in the next three figures to show that above the AH the neuron is suprathreshold, between the AH and the SNP the neuron is bistable, and below the SNP it is subthreshold. Figure 1 shows the transition from the stationary asynchronous state to the SPO. In the stationary asynchronous rate (Fig. 1*A*), the mean firing rate of the population is stable over time; an increase (decrease) from the mean firing rate recruits more (less) inhibition, which lowers (raises) the firing rate back towards the mean. At relatively high noise levels, increasing the external bias current triggered a transition through a population Hopf bifurcation to the SPO because the increased bias current increases the firing rate so much that the additional inhibition recruited overshoots the mean rate, initiating an oscillatory cycle of overcorrections (Fig. 1*B*) with small amplitude near the bifurcation. The oscillatory amplitude grows with distance from the bifurcation (Fig. 1*C*). The Hopf bifurcation for single neurons described above is unrelated to the Hopf bifurcation that occurs at a population level. However, in these subthreshold regimes, the noise overwhelms the intrinsic dynamics so that the excitability type does not matter. The transition in Figure 1 for networks of Type 2 neurons is qualitatively similar to that found for networks of Type 1 neurons. The interspike interval histogram remains approximately exponential as the drive to the network is increased (Fig. 1*A*1–*C*1), despite the emergence of an oscillation in the population rate (Fig. 1*B*2,*C*2) and in the average mean current (Fig. 1*B*4,*C*4, green curve). However, peaks at multiples of the oscillation period will always be observed if there is sufficient data and the temporal resolution is sufficiently fine.

**Figure 2.**
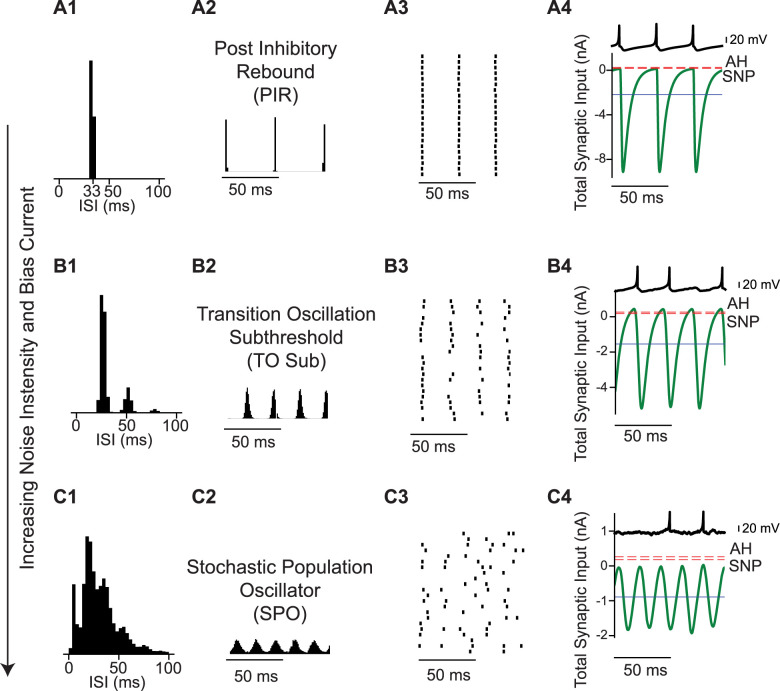
Transition from coupled oscillator synchrony to SPO in a network of Type 2 neurons with increasing bias current and noise intensity. ***A***, Coupled oscillator synchrony due to postinhibitory rebound (noise intensity is 0.03 nA and bias current is 0.2 nA). ***A*1**. ISI histogram. ***A*2**. Spike time histogram. ***A*3**. Raster plot (down sampled from 3,000 to 50). ***A*4**. Current relative to bifurcations [red dashed lines, AH Andropov-Hopf, SNP saddle node of periodics, total synaptic input current averaged over the network (green), and mean current for single neurons (dark blue line)]. Single neuron voltage trace (light blue). Spikes Per Cycle (SPC) is 1. ***B***, Gradual transition via cycle skipping (noise intensity is 0.79 nA and bias current is 0.77 nA). ***B*1–4** as in ***A*1–4**. SPC is 0.8. ***C***, With sufficient noise, the SPO emerges (noise intensity is 3.16 nA and bias current is 1.5 nA). ***C*1–4** as in ***A*1–4**. SPC is 0.58. In all the three cases the synaptic strength is 7.94 nA and the mean firing rate is 30 Hz. All units are given per cm^2^.

Figure 2 illustrates a different route to the SPO. In contrast to the transition shown in the previous figure, this transition is gradual and does not involve a bifurcation. Figure 2*A* shows a coupled oscillator regime in which the mean current is well below the threshold for repetitive firing. The concept of a spiking threshold does not strictly apply to neurons operating near a subcritical Hopf bifurcation. Between the two thresholds (AH and SNP, see methods) there is a bistable region of single neuron dynamics, in which either depolarization or hyperpolarization can trigger spiking because the stable resting potential is surrounded by an unstable limit cycle. Unlike the case for integrator neurons exhibiting Type 1 excitability (Ermentrout, 1996; Rinzel and Ermentrout, 1998; Izhikevich, 2007), an inhibitory input can trigger a spike via postinhibitory rebound (PIR), as shown in Figure 2*A*4. Here, not only does the average current remain below the bifurcations that determine the threshold for repetitive spiking, but also the instantaneous values of the total input current to a single neuron. Thus, even though this is clearly a coupled oscillator regime, the mean current is not a reliable determinant of whether the firing is “mean-driven” or fluctuation-driven. Other than the PIR nature of the coupled oscillator spiking, the transition to the SPO for networks of Type 2 neurons is similar to that found for networks of Type 1 neurons. For the coupled oscillator regime, the ISI distribution is a narrow Gaussian (Fig. 2*A*1), the population is tightly synchronized (Fig. 2*A*2,*A*3) and neurons spike on every cycle (Fig. 2*A*4). As the noise increases, neurons begin to skip cycles, leading to the transition regime in Figure 2*B*, with peaks in the ISI histogram (Fig. 2*B*1) at subharmonics of the population frequency corresponding to skipped cycles, and a subthreshold oscillation in the membrane potential of individual neurons during a skipped cycle (Fig. 2*B*4, light blue trace). Once the firing rate of individual neurons becomes sufficiently sparse, the peaks in the ISI histogram merge into a seemingly exponential distribution (Fig. 2*C*1) and firing in single neurons appears random (Fig. 2*C*2,*C*4) despite a clear oscillation in the population rate (Fig. 2*C*3). As stated above, there is no bifurcation, only a gradual transition from a coupled oscillator regime to the SPO.

**Figure 3.**
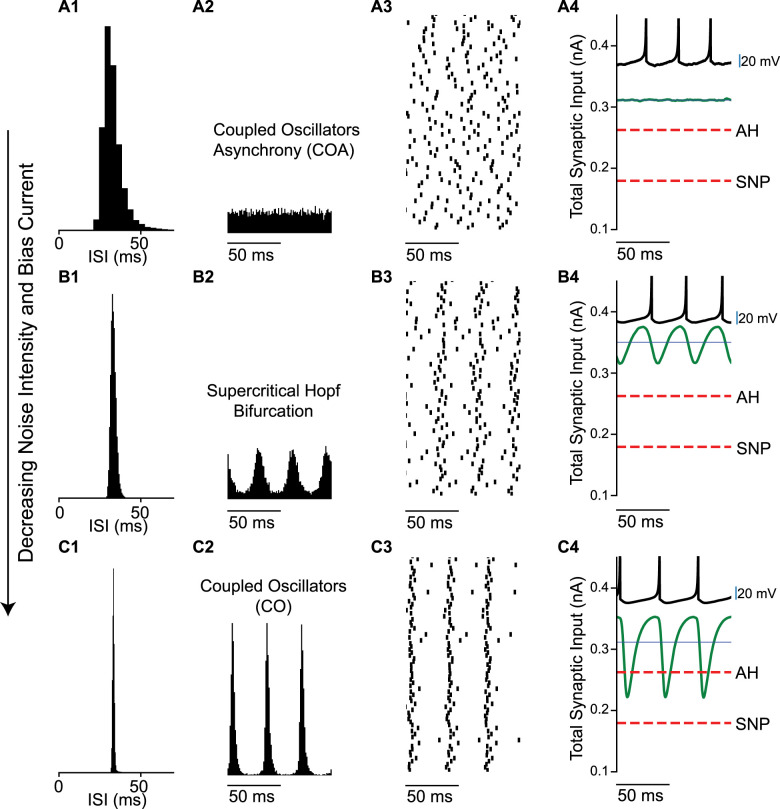
Transition from coupled oscillator asynchrony to coupled oscillator synchrony in a network of Type 2 neurons with decreasing noise intensity and bias current. ***A***, At high noise, the asynchronous state has a stationary mean firing rate (noise intensity is 0.56 nA and bias current is 0.428 nA). ***A*1**. ISI histogram. ***A*2**. Spike time histogram. ***A*3**. Raster plot (down-sampled to 71 out of 3,000 neurons). ***A*4**. Current relative to threshold. Threshold at the saddle node (SN, red dashes) bifurcation, total synaptic input current averaged over the network (green), and mean current for single neurons (blue line). ***B***, Transition from network asynchrony to synchrony (noise intensity is 0.14 nA and bias current is 0.398 nA). ***B*1–4** as in ***A*1–4**. Spikes per cycle (SPC) is 0.98. ***C***, Coupled oscillator synchrony (noise intensity is 0.05 nA and bias current is 0.359 nA). ***C*1–4** as in ***A*1–4**. SPC is 0.99. In all the three cases the synaptic strength is 0.16 nA and mean firing rate is 30 Hz. All units are per cm^2^.

**Figure 4.**
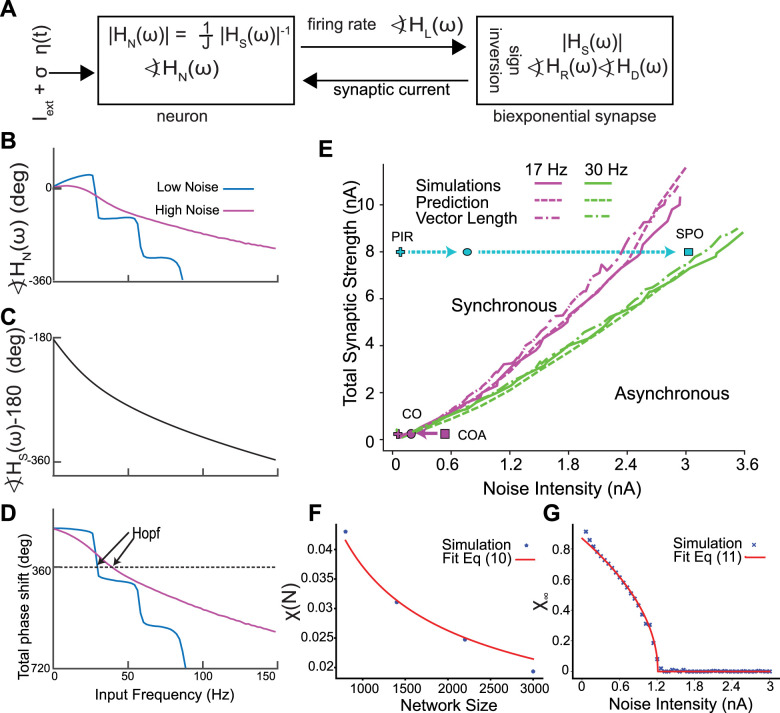
Hopf bifurcation between stochastic asynchrony and synchrony. ***A***, Self-consistent criterion for an oscillation in population rate. The sum of the phase shifts must equal −360°. ***B–D***, Self-consistent phasic relationship for an oscillation in population rate**. *B***, Numerically calculated neuronal phase shifts with σ = 0.16 nA, for low noise and σ = 2.8 nA for high noise. Iext is adjusted to maintain a constant firing rate of 30 Hz. ***C***, Analytically calculated synaptic phase shift. ***D***, The sum of the phase shifts must equal zero or a multiple of 360° (see Fig. 2) ***E***, Projection of two curves onto the noise versus synaptic strength plane. Prediction, simulation result. The points corresponding to Figures 2 and 3 are depicted in cyan and purple, respectively. Dashed arrows depict transition in the synchronous region, full arrows correspond to asynchronous region. In *E*, we vary *I*_ext_ for each ($\sigma_{C}$, *J*) to keep the mean firing rate constant. ***F, G***, Finite-size scaling to find the Hopf bifurcation for Izhikevich model. ***F***, Value of χ(N) for the four sizes of the network together with the fit of Equation 9. This fit gives a value of $\chi_{\infty }$ for each *J*, σ and *I*_ext_. In this case *J* = 2 nA, σ = 2 nA and *I*_ext _= 2 nA. ***G***, After finding $\chi_{\infty }$, we find the noise value that destabilizes the asynchronous state for each value of *J* and *I*_ext_. Thus, we end up with a set of values (*J*, *I*_ext_, $\sigma_{C}$) which determines the Hopf bifurcation surface. *J* and *I*_ext_ are as (in ***F***). All units are given per cm^2^.

Figure 3 shows the transition from asynchrony to synchrony in the coupled oscillator regime. Consistent with previous studies (Brunel and Hansel, 2006) and with Figure 1, the transition is via a supercritical Hopf bifurcation. At high levels of noise and external bias current, individual neurons fire irregularly as evidenced by the ISI histogram (Fig. 3*A*1), the firing rate is stationary over time (Fig 3*A*2) and the raster plot shows the network is desynchronized (Fig. 3*A*3). The mean synaptic current (green curve in Fig. 3*A*4) is above both thresholds for repetitive firing. At the Hopf bifurcation, determined by the mean-field analysis (Fig. 4*A*), the input to an individual neuron becomes sinusoidal (Fig. 3*B*4) and remains suprathreshold. In this case, the firing rate was held constant at 30 Hz by decreasing the noise and bias current simultaneously. Decreasing the noise allows the network to start firing in a correlated manner (Fig. 3*B*3), causing an oscillation in the population rate (Fig. 3*B*2) that regularizes the ISI histogram, which is now centered around the mean firing rate (Fig. 3*B*1). As the noise (and bias current) are decreased further, a tight global synchrony emerges (Fig. 3*C*2,*C*3) with neurons firing very regularly, evidenced by the narrow, approximately Gaussian ISI histogram (Fig. 3*C*1). The original theoretical work (Brunel and Hansel, 2006) examined a 2D parameter space of the standard deviation of the current noise and the strength of an individual current-based inhibitory synapse, converted to units of voltage by multiplying by the membrane resistance (at rest). The firingrate was kept constant by adjusting the mean level of excitatory external bias current as in Figure 3.

Figure 4 explains how the transitions between asynchrony and synchrony can be predicted using mean field theory, which assumes all neurons in the network receive identical input. The oscillations in firing rate and oscillations in synaptic current give the output and input of the network, respectively, and must be self-consistent as illustrated in Figure 4*A* and described in the Methods. $∢H_{R}(\omega )$ and $∢H_{D}(\omega )$ represent the phase lags for the rising and decaying exponentials in the definition of the synapse; $∢H_{L}(\omega )$is the phase lag for the synaptic latency; $∢H_{N}(\omega )$ is the phase lag for the neural model, while $\left|H_{S}(\omega )\right|$ is the amplitude scale factor for the biexponential synapse with unitary conductance strength.

**Figure 5.**
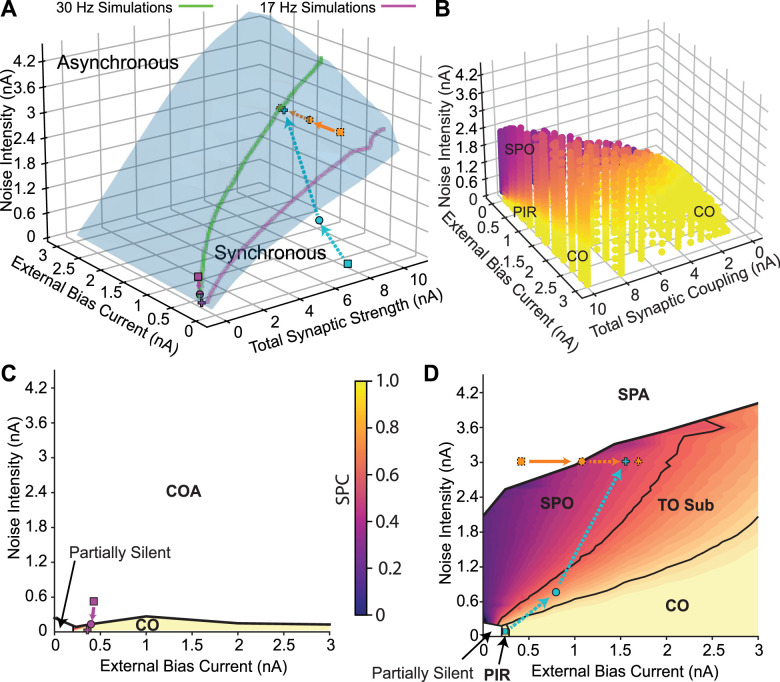
Transitions in the full 3D parameter space. ***A***, Bifurcation in the full 3D parameter space. The points corresponding to Figures 1–3 are depicted in orange, cyan and purple, respectively. Dashed arrows depict transition in the synchronous region, full arrows correspond to asynchronous region. ***B***, Transition from coupled oscillator synchrony to SPO**.** The color code indicates the spikes per cycle (SPC), which is the average fraction of oscillation cycles in which an individual neuron spikes. Population oscillations disappear on the upper surface of the colored region, which is the Hopf bifurcation surface from a different perspective than that shown (in ***A***). ***C***, Plane depicting the low coupling region of the 3D space. The transition is from coupled oscillators synchrony (CO) to coupled oscillators asynchrony (COA). Total synaptic coupling is 0.16 nA. ***D***, Plane depicting the high coupling region of the 3D space. Total synaptic coupling is 7.94 nA. All units are given per cm^2^.

Figure 4*B–D* gives an example of the determination of the location of the Hopf bifurcation that gives rise to a SPO (see Materials and Methods for detailed explanation). The neuronal phase shift for a low noise case ( Fig. 4*B*, blue curve) and a high noise case (magenta curve) was calculated numerically by applying a sinusoidal drive to a single neuron (Fig. 4*A*) with different contributions of a steady bias current and added noise to give the same mean firing rate of 30 Hz. The synaptic phase shift (Fig. 4*C*) for a biexponential synapse is the same for both cases. The 180° phase shift for the sign inversion shown in Figure 4*A* was combined with other two phase shifts in Figure 4*D*. A peak in the rate causes, with synaptic delay, a peak in the absolute value of the inhibitory synaptic current. The current then decays to a minimum. After another delay, which in is part dependent on the neuronal properties, the current minimum results in another peak in the rate. The timing of the delays between peaks (or any part of the rate waveform) must equal the cycle period for global synchrony to exist. Multiple current peaks in between rate peaks correspond to solutions in which subclusters fire one after the other in the coupled oscillator regime. Global synchrony can arise when the oscillation in rate is phase locked 1:1 with the oscillation in current and remains in phase with itself after traversing the loop in Figure 4*A*, meaning that the sum of the phase shifts equals −360°. For the high noise case, the intersection is at 46.7 Hz. For the low noise case, the intersection at 31 Hz corresponds to a Hopf bifurcation for global synchrony (the intersection at −720 is a two-cluster solution [Brunel and Hansel, 2006)]. Figure 4*E* shows a spot check of the mean field predictions (dashed curves) of the location of the Hopf bifurcation at two constant firing rates, (17 and 30 Hz), projected onto the plane of synaptic strength and standard deviation of the noise. The neuronal phase shift and the transfer function were calculated from the largest Fourier coefficient (Richardson, 2008). The mean field theory (dashed curves) accurately predicts the actual Hopf bifurcation (solid curves) in both the coupled oscillator and SPO regimes. We also utilized a vector length method (dot-dashed curves) described in the Methods to quantify the onset of synchrony at the bifurcation. The point at which the vector length exceeded 0.3 provided a reasonable approximation to the Hopf bifurcation in Figure 4*E*. The transitions in Figures 2 and 3 were generated at a constant firing rate of 30 Hz; thus they can be visualized in Figure. 4*E*. The cyan line shows the path from coupled oscillators to SPO from Figure 2; all values are to the left of the Hopf bifurcation into asynchrony shown at 30 Hz by the green curve. The purple line shows the transition in Figure 3 from a mode in which neurons are suprathreshold but asynchronous (coupled oscillator asynchrony COA) to a coupled oscillator (CO) synchronous regime when the green curve is crossed from the left. The solid curves in Figure 4*E* were obtained by applying the synchrony measure χ(N) for networks of size N in Figure 4*F* described in the Methods to find the synchronization index for an infinitely large network $\chi_{\infty }$. The point at which this index deviates from zero is the Hopf bifurcation (Fig. 4*G*). Hence this metric (as described in the Methods) was used to determine the full 3D structure of the Hopf bifurcation surface (Fig. 5*A*) that separates synchrony from asynchrony.

The transition from the asynchronous stationary state to SPO from Figure 1 is shown as an orange curve in Figure 5*A* and can also be visualized as a downward crossing of the contour of the top of the surface shown in Figure 5*B*; that manifold is the Hopf bifurcation shown at a different angle from the one in Figure 5*A*.

The transition from coupled oscillators (including the PIR regime) from Figure 2 to SPO (cyan trace in Fig. 5*A*) is gradual via cycle skipping. Figure 5*B* illustrates the gradual transition via cycle skipping shown in Figure 2 in the full 3D space as the participation index (SPC) drops from yellow for full participation in which every oscillator participates in each cycle of the population oscillation and decreases gradually (orange) as the SPO region (purple) is approached. The transition from coupled oscillator to SPO (cyan trace) is also shown in the plane section in Figure 5*D*; the PIR region is marked for low noise and low bias current, and TO stands for transitional oscillation with subharmonic peaks.

The transition from coupled oscillator asynchrony to synchrony from Figure 3 is shown in purple in Figure 5*A*. The SPC measure is only defined for synchronous regions, thus the coupled oscillator asynchrony and stochastic population asynchrony regions are not evident in Figure 5*B*. Figure 5*C* depicts a plane section of the bifurcation diagram in the low coupling region and shows the transition in Figure 3 (purple curve) from a CO asynchronous to a CO synchronous state as the Hopf bifurcation (black curve) is crossed.

### Transitions to SPO in Type 2 model with conductance-based synapses are more robust to noise with hyperpolarizing compared to shunting synapses

**Figure 6.**
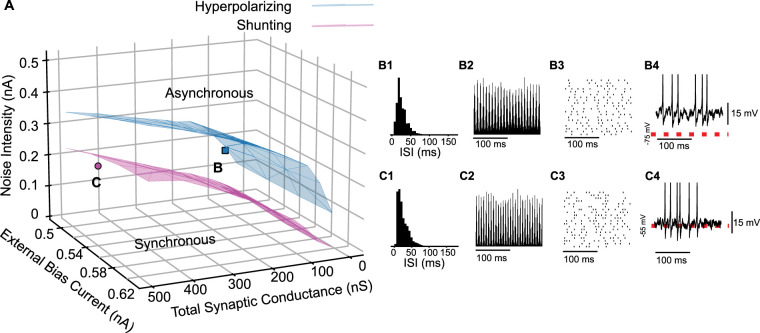
Hyperpolarizing inhibition is more robust to noise than shunting inhibition for all values of coupling and external current. ***A***, Bifurcation in the full 3D parameter space for hyperpolarizing (blue) and shunting (magenta) inhibition. ***B***, SPO for hyperpolarizing inhibition. Peak conductance is 66 nS. ***B*1**, ISI histogram. ***B*2**. Spike time histogram. ***B*3**, Raster plot (down-sampled). ***B*4**, Membrane potential of a representative neuron. ***C***, SPO for shunting inhibition. Peak conductance is 412.5 nS. ***C*1**, ISI histogram. ***C*2**, Spike time histogram. ***C*3**, Raster plot (down-sampled). ***C*4**. Membrane potential of a representative neuron. In both cases the noise intensity is 0.16 pA and bias current is 0.5 nA. Red dashed lines indicate the synaptic reversal potential.

The results in the previous figures were all obtained with current-based synapses. We used conductance-based biexponential synapses and a biologically calibrated model of the PV+ inhibitory interneurons with Type 2 excitability in layers 2/3 of the medial entorhinal cortex. Previous studies in the dentate gyrus suggested that the reversal potential of GABA_A_ synapses on PV+ fast spiking basket cells is shunting (Vida et al., 2006) rather than hyperpolarizing. However, a more recent study (Otsu et al., 2020) suggests that in area CA1 the reversal potential can be quite variable. Figure 6*A* shows the full 3D parameter space and the region of synchrony and asynchrony for both hyperpolarizing and shunting inhibition. Similar to previous results for the coupled oscillator regime (Via et al., 2022), hyperpolarizing synapses are more strongly synchronizing and are thus more robust to noise. The red dashed lines in Figure 6*B*4 and *C*4 explain the difference between hyperpolarizing and shunting synapses. Hyperpolarizing synapses cause downward deflections in the membrane potential towards the reversal potential (red dashed line in Fig. 6*B*4). In contrast, a shunting synapse does not produce appreciable deflections in the membrane potential (reversal potential indicated by red dashed line in Fig. 6*C*4), but only increases the conductance and resists deflections from that reversal potential. Figure 6*B* shows a SPO in a network with synapses with a hyperpolarizing reversal potential of −75 mV and exhibiting an approximately exponential ISI histogram (Fig. 6*B*1), an oscillation in population rate (Fig. 6*B*2), sparse firing (Fig. 6*B*3), and a random walk in the membrane potential of individual neurons (Fig. 6*B*4). We then examined the case in which the reversal potential of the inhibitory synapses was set to a shunting value of −55 mV. A SPO (Fig. 6*C*) was easily observed in this network. Therefore, the SPO does not require hyperpolarizing inhibition. Nonetheless, the shunting synapses destabilize the asynchronous regime for a lower noise level, which means that the synchrony is less robust to noise for these synapses, both in the coupled oscillator and the SPO regimes.

**Figure 7.**
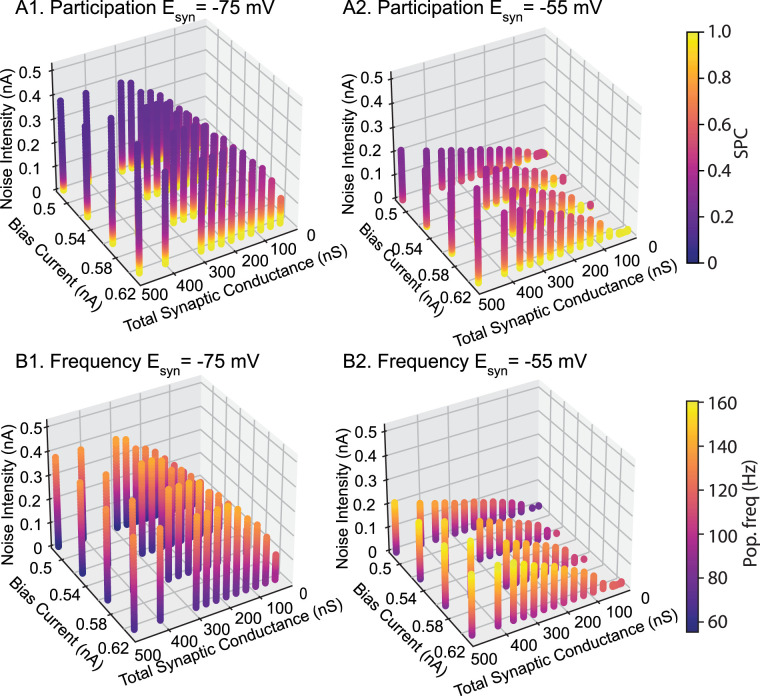
Participation and frequency in homogeneous networks of Type 2 interneurons with hyperpolarizing compared to shunting inhibition. ***A***, Participation is quantified as the fraction of the population that participates on average in each oscillation cycle, or spikes per cycle (SPC). ***B***, The oscillation frequency in general remains in the fast gamma regime.

Figure 7 shows the participation (top) and frequency for the homogeneous network with hyperpolarizing and shunting conductances. Consistent with Figure 6*A*, synchrony is more robust to noise with hyperpolarizing inhibition, as evidenced by the taller columns in A compared to B. Participation is highest with low noise and falls off as noise is increased. The prevalence of cool colors in the region in Figure 7*A*1 that supports synchrony for hyperpolarizing, but not shunting (Fig. 7*A*2), inhibition shows that synchrony is preserved via sparse participation at high noise intensity. For comparable parameter values, networks with shunting inhibition exhibit greater participation than hyperpolarizing, except at low noise values, and generally faster frequencies (Fig. 7*B*). Hyperpolarizing inhibition displaces the membrane potential and time is required for the membrane potential (and therefore the firing rate) to recover after an inhibition, lengthening the cycle period. At a given synaptic reversal potential, frequency appears to be largely independent of the conductance value and applied current value but is determined by the noise level. Figure 4*B*1 shows that increasing noise makes the dependence of the neuronal phase lag on frequency less steep, resulting in an intersection with the −360 line at higher frequencies as in Figure 4*B*3.

### Uniform distribution in reversal potential is less stable than hyperpolarizing for a homogeneous network

According to a recent study, the reversal potential of somatically evoked GABA_A_R-mediated currents for PV+ interneurons in CA1 is distributed from −55 to −75 mV, in an approximately uniform way (Otsu et al., 2020). There is also a somatic-dendritic gradient within the PV+ cells, which justifies varying the synapses projecting to a single neuron (Otsu et al., 2020). Thus, we compared networks with either homogeneously hyperpolarizing or shunting synapses to a network with uniform distribution between the two extremes. Figure 7 shows that although the hyperpolarizing synapses are still the most synchronizing ones (Fig. 7*A,B*) across all the parameter space, a uniform combination of shunting and hyperpolarizing synapses is not always more synchronizing than shunting (Fig. 7*A,B*). For a region with low total synaptic coupling (below around 2–2.5 nS) the uniform distribution of reversal potentials is more stabilizing than shunting, whereas the opposite occurs above these values (Fig. 7*B*).

#### Biophysically realistic network

**Figure 8.**
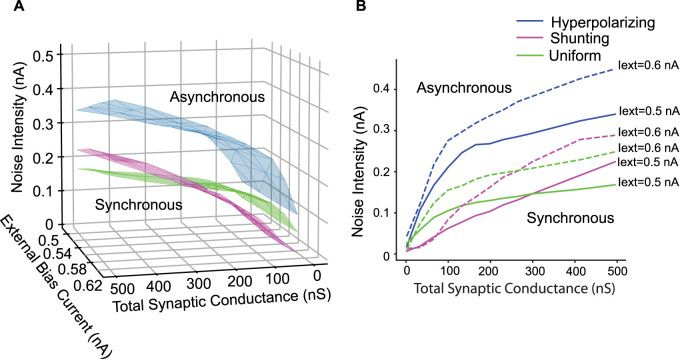
Uniform distribution of reversal potential is less robust than hyperpolarizing for all values of bias current, noise and coupling, and less robust than shunting for high synaptic coupling. ***A***, Bifurcation in the full 3D parameter space for hyperpolarizing (blue), shunting (magenta) inhibition and uniform distribution (green) for reversal potential between −55 and −75 mV. ***B***, Hopf bifurcation in the total synaptic coupling versus noise intensity for external bias current equal to 0.5 nA (full line) and 0.6 nA (dashed line) for hyperpolarizing (blue), shunting (magenta) and uniform distribution (green). For synaptic coupling higher than 250 nS (for 0.6 pA of bias current) and 300 nS (for 0.5 pA), a uniform distribution in synaptic reversal potential is less robust than shunting reversal potential.

**Figure 9.**
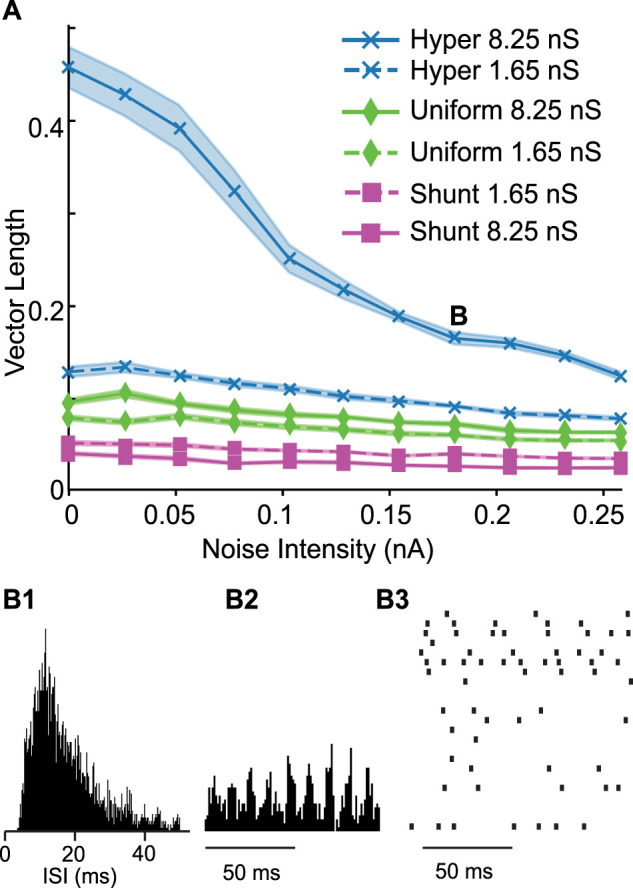
Comparison of different kinds of inhibition in a heterogeneous network. ***A***, Average vector length at different noise levels. Shaded area shows the standard error of the mean. Dashed lines are for the weaker conductance value. The labels are in the same order as the traces. ***B***, SPO. ***B*1**, ISI histogram. ***B*2**, Spike time histogram. ***B*3**, Down-sampled raster.

The results in Figures 4–8 were obtained for homogeneous networks because their properties can be analyzed precisely in the limit of an infinite network. In order to show that the same general principle applies to a biophysically realistic network, we constructed a model of layer 2/3 PV+ interneurons that captures the measured heterogeneity in the electrophysiological properties of these neurons (Via et al., 2022) as well as the variability in synaptic connectivity. Both connection probability and connection strength were randomized as described in the Methods section on the *Heterogeneous Network*. We ran ten simulations at each noise value for the measured average synaptic conductance value (1.65 nS) and for an average five times larger (8.25 nS) to ensure that we sampled the SPO regime (point B in Fig. 9*A* with the ISI distribution, population spike time histogram and down-sampled raster plot in Fig. 9*B*1–3). For each of the two conductance values, hyperpolarizing inhibition was the most strongly synchronizing in the presence of noise, whereas shunting inhibition was the least effective, with a uniform distribution at an intermediate level of synchrony between the two extremes. Increasing the conductance for hyperpolarizing and uniformly distributed conductances always increased the vector length, whereas for shunting it decreased the vector length. A Kruskal–Wallis test showed that the medians of the distributions at each noise and conductance level differed in every case for the three types of inhibition with a *p* < 4 × 10^−6^, and a post hoc pairwise Wilcoxon test showed pairwise differences in every case with *p* < 4 × 10^−05^.

## Discussion

### Type 2 networks with current-based synapses

The transitions in Figures 1 and 3 cross the border between asynchrony and synchrony to the SPO regime and to the coupled oscillator regime, respectively, and are equally well predicted by mean field theory (Fig. 4*E*). Another transition does not involve a bifurcation but instead involves a gradual increase in cycle skipping by which coupled oscillator neurons that fire early in a cycle suppress some of the other neurons on that cycle (Fig. 2), until the inhibition is so strong that neurons participate only sparsely and apparently randomly. A somewhat counterintuitive finding is that in “mean-driven” coupled oscillator regimes, the mean current can be subthreshold (Fig. 2*A*). The bifurcations and gradual transitions observed in networks of inhibitory neurons with Type 2 excitability parallel those in networks with Type 1 excitability (Brunel and Hansel, 2006).

### Networks with conductance-based synapses

The transitions described in the preceding paragraph for current-based synapses generalize to conductance-based synapses. The representation of an inhibitory synapse by an outward current waveform implicitly assumes a hyperpolarizing synapse. Using conductance-based synapses introduces an additional parameter, that of the synaptic reversal potential, which is neglected in the studies with current based synapses. Although shunting synapses do not carry the hyperpolarizing currents upon which the SPO theory is based, networks with shunting synapses nonetheless exhibited SPOs (Fig. 6*C*). Moreover, the synaptic reversal potential parameter *E*_GABA,A_ exerts a powerful influence on synchronizing tendencies, consistent with previous studies (Wang and Buzsáki, 1996; Proddutur et al., 2013). In coupled oscillator networks, we have shown that the phase response curves (PRC) of experimentally calibrated models of PV+ interneurons explain why hyperpolarizing synapses are more strongly synchronizing than shunting synapses (Via et al., 2022) (provided there are synaptic delays in the physiological range of 1 ms), in contrast to a previous study (Vida et al., 2006). Specifically, if all other parameters are held constant at sufficiently strong synaptic conductance value, the PRC for hyperpolarizing inhibition exhibits monotonically increasing delays with a slope of one, whereas the PRC for shunting inhibition is rather flat (Via et al., 2022). The slope of the PRC at the locking point determines how quickly perturbations from synchrony decay or grow, and the PRC for strong hyperpolarizing is maximally stabilizing. Intuitively, the inhibition brings the neuron to equilibrium near the synaptic reversal potential such that the memory of the previous phase is erased, and there is a constant rebound interval until the next spike. This results in a linear PRC with a slope of one, which, as stated above, is maximally stabilizing. The present study extends these results that hyperpolarizing synapses are more strongly synchronizing to the SPO regime. We hypothesize that the synchronizing tendencies in the coupled oscillator regime persist in the SPO regime for Type 2 neurons because their resonant properties endow them with a preferred frequency (near the minimum frequency that can sustain repetitive spiking) even in the subthreshold regime. Under this scenario, the synchronizing tendencies of their PRCs are still relevant.

The blue Hopf bifurcation curves in Figure 8*B* for hyperpolarizing synapses have an inflection point at which the slope flattens out. We suspect that this is due to the saturating effect of hyperpolarizing conductance-based synapses. Whereas outward current synapses can cause an unlimited amount of hyperpolarization, conductance-based synapses cannot hyperpolarize the membrane potential beyond the reversal potential (−75 mV here), and therefore saturate. The green curves for a uniform distribution of firing rates also have an inflection point, but it occurs at higher values of conductance strength and is not as pronounced, likely due to the presence of shunting synapses as well as hyperpolarizing. The red curves for shunting inhibition (−55 mV) do not have an inflection point in the regime studied and have a slope intermediate between the steep and flat portions of the green curve, which results in the intersection points in which robustness of these two synaptic distributions to noise is reversed. At high levels of bias current, the membrane potential of individual neurons in the model may become sufficiently depolarized that synapses with a reversal potential of −55 mV have a small hyperpolarizing effect, which could be a potentially nonphysiological confound in our analysis.

#### Heterogeneity

Previous theoretical studies used temporal heterogeneity in that individual neurons receive distinct current noise, as we did in Figures 4–8. Actual inhibitory interneurons are heterogeneous in the synaptic connectivity, their passive and active properties (Fernandez et al., 2022; Via et al., 2022). We honored this heterogeneity in Figure 9 and found that hyperpolarizing inhibition rendered synchrony in the heterogeneous network more robust to noise. This builds upon our earlier results that hyperpolarizing inhibition is better at synchronizing heterogeneous inhibitory interneurons in the coupled oscillator regime. Modeling the dendritic locus of inhibitory synaptic input makes interneurons more robust to heterogeneity (Kriener et al., 2022).

#### Increasing gamma synchrony as therapeutic strategy

PV+ fast spiking inhibitory interneurons have been shown to mediate gamma synchrony (Bartos et al., 2007; Cardin et al., 2009; Sohal, 2022). In some cases, the gamma oscillations can be supported by inhibitory interneurons alone (Vinck et al., 2013, 2015, 2016) as in the computational examples in the current study, although there is frequently an interplay between inhibitory and excitatory cells (Tiesinga and Sejnowski, 2009). There is mounting evidence to suggest that enhancing gamma synchrony may have multiple therapeutic benefits. Optogenetic stimulation of entorhinal cortex perforant path engram cells at high-gamma (100 Hz) frequency rescued memory impairments in a mouse model of AD (Roy et al., 2016). Moreover, gamma oscillations can also affect molecular pathology; entrainment of gamma in the visual cortex by 40 Hz flickering light and by optogenetic stimulation of PV+ interneurons in hippocampal area CA1 in a mouse model of AD reduces Aβ levels in those respective areas (Iaccarino et al., 2016).

In a coupled oscillator regime, enhancing gamma synchrony could involve targeting specific intrinsic ion channels to fine tune the phase resetting properties. Regardless of the oscillatory mechanism, this study shows that the reversal potential for GABA_A_ receptor chloride channels is a likely therapeutic target to enhance gamma synchrony. However, it seems likely that the synaptic reversal potential is heterogeneous across synapses (Blaesse et al., 2009; Otsu et al., 2020). If we assume a physiological network with the synaptic reversal potential distributed uniformly between the two extreme values, then manipulations to reduce internal Cl^−^ concentrations in PV+ interneurons should increase gamma synchrony, because networks with purely hyperpolarizing synapses synchronize more robustly than those with a uniform distribution (Fig. 8).

#### Implementation of therapeutic strategies

In mature neurons, the reversal potential of the GABA_A_ synapses is thought to be determined by the expression of anion cotransporters, primarily KCC2 (Blaesse et al., 2009). However, bicarbonate ions also flow through GABA_A_ channels, causing the reversal potential of GABA_A_ to be more depolarized than the reversal potential for chloride *E*_Cl_ (Blaesse et al., 2009). One caveat in designing therapies to modify *E*_Cl_ is that chloride microdomains due to the inhomogeneous distribution of anionic polymers such as actin, tubulin, and nucleic acids (Rahmati et al., 2021) may exert a strong local influence on *E*_Cl_. There is growing evidence that a defective ratio of chloride importer NKCC1 and chloride exporter KCC2 is present in several neurodevelopmental conditions (Savardi et al., 2021). Since these cotransporters are fundamental in the regulation of neuronal chloride concentration, therapies focused on restoring chloride homeostasis, and hence modifying *E*_GABA,A_, could have an impact in restoring core symptoms of several disorders. For example, (Parrini et al., 2021) showed that reducing NKCC1 expression in a Ts65Dn mouse model of Down syndrome restores the intracellular chloride concentration, efficacy of GABA-mediated inhibition, and neuronal networks dynamics, rescuing cognitive deficits as well. Thus, although there are many technical challenges to selectively targeting the reversal potential for GABA_A_ synapses selectively in PV+ inhibitory interneurons, this strategy could have powerful implications for gamma synchrony and its potential role in reversing cognitive impairment.

## Synthesis

Reviewing Editor: Arvind Kumar, KTH Royal Institute of Technology Decisions are customarily a result of the Reviewing Editor and the peer reviewers coming together and discussing their recommendations until a consensus is reached. When revisions are invited, a fact-based synthesis statement explaining their decision and outlining what is needed to prepare a revision will be listed below. The following reviewer(s) agreed to reveal their identity: Charles Wilson. Note: If this manuscript was transferred from JNeurosci and a decision was made to accept the manuscript without peer review, a brief statement to this effect will instead be what is listed below.

Synthesis

In the paper authors have studied the dynamics of population oscillation in a fully connected network of type-2 neurons. Both the reviewers agreed that the work is done rigorously and the finding that hyperpolarizing inhibition is more effective than conductance change is interesting but perhaps expected already from the work of Brunel and colleagues.

Nevertheless the reviewers think that even if idealized the results could be useful. However, it is important to show that the results obtained from an idealized network model also extend to network models that are more close to biology. Fully connected networks of type-2 neurons are not found anywhere. So studying the phenomenon in a more biologically motivated model is essentially - since the authors focus a lot on gamma band oscillations and synchrony they should consider a model based on the known architecture of the neocortex or hippocampus. Without that the paper does not really fit within the scope of eNEURO.

Reviewer 1

The paper extends the theory of stochastic and coupled population oscillators by investigating the population dynamics of type II inhibitory neurons. These are typically called "resonators" (per Izhikevich's classification) and are characterized by transition into spiking activity through a supercritical Hopf-bifurcation that keeps the firing rate above of a lower non-zero bound (i.e., there is a discontinuous jump in the f-I curve). Previous works that are herein extended have analyzed similar questions but in "integrators" (type I inhibitory neurons in which firing occurs through a saddle-node on invariant circle bifurcation, with frequency starting near zero).

I found the paper very-well written. I think it covers some interesting questions about the transition of populations of inhibitory neurons from an asynchronous to the synchronous state. It provides a systematic, in-depth analysis of how the network dynamics changes as a function of three (experimentally relevant) parameters: noise intensity, external bias current, and total synaptic coupling (or synaptic conductance, respectively). The analysis is done numerically by constructing explanatory bifurcation diagrams and appears to be sound.

Some of the strengths are:

(1) providing a quantitative understanding of how networks of type-II inhibitory neurons may transition between states such as asynchronous firing to stochastic population oscillators.

(2) comparison of two distinct conditions, when the network's units are either intrinsic oscillators (the coupled oscillators case/ mean-driven stochastic population oscillation) or when the stochastic oscillations result in the network by destabilization of the units' stationary background firing rate (fluctuation-driven);

(3) implantation of more realistic conductance-based synapses. This approach led to some meaningful predictions on the network's dynamics and robustness of synchrony, based on changes in the synaptic reversal potential for the GABA-A synapses (from shunting inhibition at about -55 mV to hyperpolarizing state at about -75 mV).

I only have some minor, typo-related comments to make, but otherwise I am positive about the paper and support its publication in eNeuro.

Page 20: line 15. After the word "Methods":

--- for clarity, please add an explanation of the notation from the figure (even if you have already given the details in methods), i.e., say for example, that H_R(\omega), H_D(\omega) represent the phase lags for the rising and decaying exponentials in the definition of the synapse; H_L(\omega) is the phase lag for the synaptic latency; H_N is the phase lag for the neural model, while |H_S| is the amplitude scale factor for the biexponential synapse with unitary conductance strength.

Minor corrections/typos:

Page 6, right after eq (3): there is a reference to I_{app} which has not been shown yet.

Page 6: last two lines: g_{Kv1} and g_{KV7}: it was stated above that Kv7 was mislabeled in a previous publication, plus in the current equations form the previous line there is no g_{Kv1}. Which one is it?

Page 8: the lines above Eq(6): "rhs"-should be written as "right-hand side"

Page 9 - the 2nd line from the bottom: add "H" in

"[...] but the transfer function H must [...]"

Page 10: line 6 at the end: "and" - "an"

Page 11: the paragraph that starts with "Synchrony measure": cut one copy of "the boundary"

Page 19: Figure 3-axes in panels 4: sinaptic -> synaptic. Also in the caption, for B: maybe should change the order so it reads "synchrony to asynchrony"

Page 21: caption of Figure 4: "desynchronizes the asynchronous state" ???

Page 25: caption Fig 6: last four lines-- Bs should be Cs

Reviewer 2

This computational modeling study describes the emergence of population oscillations in a fully connected network of resonant inhibitory neurons.

The network being studied is highly idealized, and networks like this (fully and equally connected pure networks of identical instances of a single cell type) never occur in nature, but the paper has some application. It is valuable to understand the relatively simple behavior of highly idealized networks as a stepping-stone toward developing intuition about realistic networks. Additionally, there is some evidence that resonant fast-spiking cortical interneurons may interact among themselves to generate some features of cortical oscillations. The intuition obtained by purified models like this could be useful in understanding that phenomenon. I think the work was careful and rigorous but the scope of it was small compared with other papers of this type.

The paper is based on some previous studies of similarly idealized networks of integrative (non-resonant) neurons, and the results are best understood in the comparison with that network. It would have been easier for the reader if some of the results of those studies had been replicated for comparison with the current results. It really was necessary for me to find those papers and open them up and do the comparison as I read this one. This paper actually addresses only a fraction of the dynamical properties that were studied in those papers. Especially missing was a discussion of how the frequency of the population oscillation is determined. In these simulations, the oscillation was in or close to the gamma range, but it was not at all clear why what was, or whether it was necessarily always so. There was some substantial change in oscillation frequency across the examples in Figures 1-3.

The main finding, highlighted in the title, is that hyperpolarizing synapses are more effective in producing network oscillations than pure conductance changes that do not alter the membrane potential. This is clear from the figures, but I'm unclear about what the authors think is the mechanism of this difference. Does the membrane potential change itself have an effect on synchronization? I was left wondering what to think about this clear fact.

A substantial portion of the discussion is spent speculating about gamma synchrony in the cortex, and the possible benefits of enhancing it for therapeutic purposes. The authors suggest that it may be possible to alter the GABAA reversal potential to enhance synchrony. Because inhibitory transmission also has a large effect on overall firing rate, it seems likely that any substantial change in reversal potential would do much more than alter synchrony.

## Author Response

Response to Reviewers We thank the reviewers and the editor for their thoughtful comments, we think the manuscript is much improved due to their critiques.

Synthesis 1) In the paper authors have studied the dynamics of population oscillation in a fully connected network of type-2 neurons. Both the reviewers agreed that the work is done rigorously and the finding that hyperpolarizing inhibition is more effective than conductance change is interesting but perhaps expected already from the work of Brunel and colleagues.

Thank you for these comments.

2) Nevertheless the reviewers think that even if idealized the results could be useful. However, it is important to show that the results obtained from an idealized network model also extend to network models that are more close to biology. Fully connected networks of type-2 neurons are not found anywhere. So studying the phenomenon in a more biologically motivated model is essential - since the authors focus a lot on gamma band oscillations and synchrony they should consider a model based on the known architecture of the neocortex or hippocampus. Without that the paper does not really fit within the scope of eNEURO.

We have extended our results to a model of an interneuronal network in layer 2/3 of the medial entorhinal cortex with heterogeneity in the active and passive properties of the model neurons as well as probabilistic connectivity with variable conductance strengths. All these properties were calibrated based on published data from our collaborators (Fernandez et al., 2022; Via et al., 2022a). New Figure 9 in the manuscript shows that the superior synchronization properties of hyperpolarizing compared to shunting inhibition are preserved in this finite, biophysically realistic network of model neurons with Hodgkin's type 2 excitability.

This new methods section describes model calibration: "Heterogeneous Network A biophysically realistic heterogeneous network was constructed only for the conductance-based Via model with a 3-D spatial structure corresponding to a mouse brain slice of layer 2/3 medial entorhinal cortex (mEC) measuring 800 µm x 300 µm x 800 µm. Based on the measured PV+ cell density in layers 2/3 of mEC (Bjerke et al., 2021), we assigned 321 neurons to the slice and positioned them randomly. The full extent of the experimentally measured variability across the PV+ population in terms of their passive properties and their frequency current relations was captured in our previously published 100 neuron model (Via et al., 2022b). We used 50 unique neurons and cloned them as necessary to construct the 321 neuron heterogeneous network. Gabaergic synaptic connectivity was calibrated according to (Fernandez et al., 2022) in which the connection probability was found to be 36% in each direction inside a radius of 150 µm with a lognormal distribution of the synaptic conductances with a of mean J*1.65 nS (Via et al., 2022b), where 1.65 nS was the measured average synaptic conductance. The standard deviation 1.56 nS of the synaptic conductances used here was less than that used in (Via et al., 2022b). However, that study assumed strong electrical synapses in order to obtain synchronous activity, and we neglect electrical synapses here. Synaptic delays were assigned with the observed range (Fernandez et al., 2022) and depend linearly on the distance inside a 150 µm radius, from 0.4 ms to 1.2 ms." In order to quantify synchrony in a finite, heterogeneous network, we introduced a vector length method: "We contrasted this method of quantifying synchrony with one developed by our group based on the cycle by cycle population period, as defined by the peaks of the population histogram (Tikidji-Hamburyan et al., 2015) and described above in the Participation Measure section. Peaks were detected using downward zero crossings of the first derivative of the smoothed signal. The peak at the beginning of each network cycle was assigned a phase value of 0 and, at the end, a phase value of 2π. Each spike was assigned a phase depending upon where it fell within the cycle and a vector length of 1 starting from the origin. Then the vector sum of all the spike vectors constructed in this fashion was taken and normalized by the number of vectors, resulting in a vector with an average phase and a length between 0 and 1. This measure quantifies the level of synchrony of the individual neurons with the population rhythm but gives no information regarding the average level of participation in the oscillation, which we track separately as the average number of spikes per cycle (SPC) normalized by the population size. Note that random peaks of spike rate can be detected in finite networks even in the absence of network oscillations, for example, in random firing or in phase dispersion. In this case, the vector length is close to 0, but the SPC still reflects the mean firing rate of the population. The advantage of the vector length measure is that only the population raster plot is required to calculate synchrony and participation therefore we used this measure for the heterogenous, finite size network. " Here are the results for the biophysically realistic network: "The results in Figs. 4-8 were obtained for homogeneous networks because their properties can be analyzed precisely in the limit of an infinite network. In order to show that the same general principle applies to a biophysically realistic network, we constructed a model of layer 2/3 PV+ interneurons that captures the measured heterogeneity in the electrophysiological properties of these neurons (Via et al., 2022b) as well as the variability in synaptic connectivity. Both connection probability and connection strength were randomized as described in the Methods section on the Heterogeneous Network. We ran ten simulations at each noise value for the measured average synaptic conductance value (1.65 nS) and for an average five times larger (8.25 nS) to ensure that we sampled the stochastic population oscillator regime (point B in Fig. 9A with the ISI distribution, population spike time histogram and down-sampled raster plot in Fig. 9B1-3). For each of the two conductance values, hyperpolarizing inhibition was the most strongly synchronizing in the presence of noise, whereas shunting inhibition was the least effective, with a uniform distribution at an intermediate level of synchrony between the two extremes. Increasing the conductance for hyperpolarizing and uniformly distributed conductances always increased the vector length, whereas for shunting it decreased the vector length. A Kruskal-Wallis test showed that the medians of the distributions at each noise and conductance level differed in every case for the three types of inhibition with a p < 4e-06, and a posthoc pairwise Wilcoxon test showed pairwise differences in every case with p<4e-05. " Next we rewrote the paragraph in the discussion covering the effects of heterogeneity: "Previous theoretical studies used temporal heterogeneity in that individual neurons receive distinct current noise, as we did in Fig. 4-8. Actual inhibitory interneurons are heterogeneous in the synaptic connectivity, their passive and active properties (Fernandez et al., 2022; Via et al., 2022b). We honored this heterogeneity in Fig. 9 and found that hyperpolarizing inhibition rendered synchrony in the heterogeneous network more robust to noise. This builds upon our earlier results that hyperpolarizing inhibition is better at synchronizing heterogeneous inhibitory interneurons in the coupled oscillator regime. Modeling the dendritic locus of inhibitory synaptic input makes interneurons more robust to heterogeneity (Kriener et al., 2022). " Reviewer 1 I only have some minor, typo-related comments to make, but otherwise I am positive about the paper and support its publication in eNeuro.

Page 20: line 15. After the word "Methods":

--- for clarity, please add an explanation of the notation from the figure (even if you have already given the details in methods), i.e., say for example, that H_R(\omega), H_D(\omega) represent the phase lags for the rising and decaying exponentials in the definition of the synapse; H_L(\omega) is the phase lag for the synaptic latency; H_N is the phase lag for the neural model, while |H_S| is the amplitude scale factor for the biexponential synapse with unitary conductance strength.

Thank you for this suggestion, we have implemented it verbatim, you can find it now at the bottom of page 22.

Minor corrections/typos:

Page 6, right after eq (3): there is a reference to I_{app} which has not been shown yet.

Iapp was changed to I(t) in this context, see next point.

Page 6: last two lines: g_{Kv1} and g_{KV7}: it was stated above that Kv7 was mislabeled in a previous publication, plus in the current equations form the previous line there is no g_{Kv1}. Which one is it? We clarified by rewriting the sentences as follows: "We included two delayed rectifier K+ currents (IKv7 and IKv3). KV7 was mislabeled as KV1 in (Via et al., 2022b). The differential equation for the membrane potential (V) of each neuron is with this correction is now:" Page 8: the lines above Eq(6): "rhs"-should be written as "right-hand side" Done.

Page 9 - the 2nd line from the bottom: add "H" in "[...] but the transfer function H must [...]" Done.

Page 10: line 6 at the end: "and" - "an" Done.

Page 11: the paragraph that starts with "Synchrony measure": cut one copy of "the boundary" Done, the sentence is now on page 13.

Page 19: Figure 3-axes in panels 4: sinaptic -> synaptic.

Done.

Also in the caption, for B: maybe should change the order so it reads "synchrony to asynchrony" Done.

Page 21: caption of Figure 4: "desynchronizes the asynchronous state" ??? "Desynchronizes" was corrected to "destabilizes".

Page 25: caption Fig 6: last four lines-- Bs should be Cs Done.

Reviewer 2 1) I think the work was careful and rigorous but the scope of it was small compared with other papers of this type.

We have increased the scope somewhat by adding a heterogeneous, biophysically realistic network as described in the response to the synthesis. We also added a figure showing how participation and frequency vary across the parameter space.

2) The paper is based on some previous studies of similarly idealized networks of integrative (non-resonant) neurons, and the results are best understood in the comparison with that network. It would have been easier for the reader if some of the results of those studies had been replicated for comparison with the current results. It really was necessary for me to find those papers and open them up and do the comparison as I read this one. This paper actually addresses only a fraction of the dynamical properties that were studied in those papers. Especially missing was a discussion of how the frequency of the population oscillation is determined. In these simulations, the oscillation was in or close to the gamma range, but it was not at all clear why what was, or whether it was necessarily always so. There was some substantial change in oscillation frequency across the examples in Figures 1-3.

We apologize for not making it clear that Figure 4 shows how the frequency is determined using the method from (Brunel and Hansel, 2006). Essentially, the delay (phase lag) between a change in the firing and when the effect of that change is able to affect the the firing rate via changes in the inhibitory synaptic current must equal a cycle period. We changed the wording on page 21 describing Fig. 4 as follows: " A peak in the rate causes, with synaptic delay, a peak in the absolute value of the inhibitory synaptic current. The current then decays to a minimum. After another delay, which in is part dependent on the neuronal properties, the current minimum results in another peak in the rate. The timing of the delays between peaks (or any part of the rate waveform) must equal the cycle period for global synchrony to exist. Multiple current peaks in between rate peaks correspond to solutions in which subclusters fire one after the other in the coupled oscillator regime. Global synchrony can arise when the oscillation in rate is phase locked 1:1 with the oscillation in current and remains in phase with itself after traversing the loop in Fig. 4A, meaning that the sum of the phase shifts equals -360o" See also the response to point 4 for a further discussion of how the frequency is set.

3) The main finding, highlighted in the title, is that hyperpolarizing synapses are more effective in producing network oscillations than pure conductance changes that do not alter the membrane potential. This is clear from the figures, but I'm unclear about what the authors think is the mechanism of this difference. Does the membrane potential change itself have an effect on synchronization? I was left wondering what to think about this clear fact.

The common hyperpolarization induced in the population with hyperpolarizing synapses is indeed synchronizing. We now cite our earlier paper that provides an explanation in terms of the phase response curves in response to hyperpolarizing versus shunting inhibition. The Discussion was modified as follows: "Moreover, the synaptic reversal potential parameter EGABA,A exerts a powerful influence on synchronizing tendencies, consistent with previous studies (Wang and Buzsáki, 1996; Proddutur et al., 2013). In coupled oscillator networks, we have shown that the phase response curves (PRC) of experimentally calibrated models of PV+ interneurons explain why hyperpolarizing synapses are more strongly synchronizing than shunting synapses (Via et al., 2022b) (provided there are synaptic delays in the physiological range of 1 ms), in contrast to a previous study (Vida et al., 2006). Specifically, if all other parameters are held constant at sufficiently strong synaptic conductance value, the PRC for hyperpolarizing inhibition exhibits monotonically increasing delays with a slope of one, whereas the PRC for shunting inhibition is rather flat (Via et al., 2022b). The slope of the PRC at the locking point determines how quickly perturbations from synchrony decay or grow, the PRC for strong hyperpolarizing is maximally stabilizing. Intuitively, the inhibition brings the neuron to an equilibrium near the synaptic reversal potential such that the memory of the previous phase is erased, and there is a constant rebound interval until the next spike. This results in a linear PRC with a slope of one, which, as stated above, is maximally stabilizing. The present study extends these results that hyperpolarizing synapses are more strongly synchronizing to the stochastic population oscillator regime. We hypothesize that the synchronizing tendencies in the coupled oscillator regime persist in the stochastic population oscillator regime for type 2 neurons because their resonant properties endow them with a preferred frequency (near the minimum frequency that can sustain repetitive spiking) even in the subthreshold regime. Under this scenario, the synchronizing tendencies of their PRCs are still relevant." 4) A substantial portion of the discussion is spent speculating about gamma synchrony in the cortex, and the possible benefits of enhancing it for therapeutic purposes. The authors suggest that it may be possible to alter the GABAA reversal potential to enhance synchrony. Because inhibitory transmission also has a large effect on overall firing rate, it seems likely that any substantial change in reversal potential would do much more than alter synchrony.

We added the following text and figure to address changes in frequency and participation: "Figure 7 shows the participation (top) and frequency for the homogeneous network with hyperpolarizing and shunting conductances. Consistent with Fig. 6A, synchrony is more robust to noise with hyperpolarizing inhibition, as evidenced by the taller columns in A compared to B. Participation is highest with low noise and falls off as noise is increased. The prevalence of cool colors in the region in Fig. 7A1 that supports synchrony for hyperpolarizing, but not shunting (Fig. 7A2), inhibition shows that synchrony is preserved via sparse participation at high noise intensity. For comparable parameter values, networks with shunting inhibition exhibit greater participation than hyperpolarizing, except at low noise values, and generally faster frequencies (Fig. 7B). Hyperpolarizing inhibition displaces the membrane potential and time is required for the membrane potential (and therefore the firing rate) to recover after an inhibition, lengthening the cycle period. At a given synaptic reversal potential, frequency appears to be largely independent of the conductance value and applied current value but is determined by the noise level. Figure 4B1 shows that increasing noise makes the dependence of the neuronal phase lag on frequency less steep, resulting in an intersection with the -360 line at higher frequencies as in Fig. 4B3." We also added a caveat to the Discussion section on Increasing Gamma Synchrony as Therapeutic Strategy "The frequency of gamma oscillations may also be affected by these manipulations." Bjerke IE, Yates SC, Laja A, Witter MP, Puchades MA, Bjaalie JG, Leergaard TB (2021) Densities and numbers of calbindin and parvalbumin positive neurons across the rat and mouse brain. iScience 24:101906.

Brunel N, Hansel D (2006) How Noise Affects the Synchronization Properties of Recurrent Networks of Inhibitory Neurons. Neural Comput 18:1066-1110.

Fernandez FR, Via G, Canavier CC, White JA (2022) Kinetics and Connectivity Properties of Parvalbumin- and Somatostatin-Positive Inhibition in Layer 2/3 Medial Entorhinal Cortex. eNeuro 9:ENEURO.0441-21.2022.

Kriener B, Hu H, Vervaeke K (2022) Parvalbumin interneuron dendrites enhance gamma oscillations. Cell Rep 39:110948.

Proddutur A, Yu J, Elgammal FS, Santhakumar V (2013) Seizure-induced alterations in fast-spiking basket cell GABA currents modulate frequency and coherence of gamma oscillation in network simulations. Chaos 23:046109.

Tikidji-Hamburyan RA, Martínez JJ, White JA, Canavier CC (2015) Resonant interneurons can increase robustness of gamma oscillations. J Neurosci 35:15682-15695.

Via G, Baravalle R, Fernandez FR, White JA, Canavier CC (2022a) Interneuronal network model of theta-nested fast oscillations predicts differential effects of heterogeneity, gap junctions and short term depression for hyperpolarizing versus shunting inhibition. PLoS Comput Biol 18:e1010094.

Via G, Baravalle R, Fernandez FR, White JA, Canavier CC (2022b) Interneuronal network model of theta-nested fast oscillations predicts differential effects of heterogeneity, gap junctions and short term depression for hyperpolarizing versus shunting inhibition Rubin J, ed. PLOS Comput Biol 18:e1010094.

Vida I, Bartos M, Jonas P (2006) Shunting inhibition improves robustness of gamma oscillations in hippocampal interneuron networks by homogenizing firing rates. Neuron 49:107-117.

Wang XJ, Buzsáki G (1996) Gamma oscillation by synaptic inhibition in a hippocampal interneuronal network model. J Neurosci 16:6402-6413.
